# Detrimental effect of the 6 His C-terminal tag on YedY enzymatic activity and influence of the TAT signal sequence on YedY synthesis

**DOI:** 10.1186/1471-2091-14-28

**Published:** 2013-11-01

**Authors:** Monique Sabaty, Sandrine Grosse, Geraldine Adryanczyk, Séverine Boiry, Frédéric Biaso, Pascal Arnoux, David Pignol

**Affiliations:** 1 CEA, IBEB, Laboratoire de Bioénergétique Cellulaire, Saint-Paul-lez-Durance F-13108, France; 2 CNRS, UMR Biologie Végétale & Microbiologie Environnementales, Saint-Paul-lez-Durance F-13108, France; 3 Aix-Marseille Université, Saint-Paul-lez-Durance F-13108, France; 4 Unité de Bioénergétique et Ingénierie des Protéines, UMR 7281, Institut de Microbiologie de la Méditerranée, CNRS, and Aix-Marseille Université, 31 Chemin Joseph Aiguier, 13402 Marseille Cedex 20, France

**Keywords:** Molybdoenzyme, YedY, TAT machinery, Signal sequence, DMSO reductase, *Rhodobacter sphaeroides*, Enzyme maturation

## Abstract

**Background:**

YedY, a molybdoenzyme belonging to the sulfite oxidase family, is found in most Gram-negative bacteria. It contains a twin-arginine signal sequence that is cleaved after its translocation into the periplasm. Despite a weak reductase activity with substrates such as dimethyl sulfoxide or trimethylamine N-oxide, its natural substrate and its role in the cell remain unknown. Although sequence conservation of the YedY family displays a strictly conserved hydrophobic C-terminal residue, all known studies on *Escherichia coli* YedY have been performed with an enzyme containing a 6 histidine-tag at the C-terminus which could hamper enzyme activity.

**Results:**

In this study, we demonstrate that the tag fused to the C-terminus of *Rhodobacter sphaeroides* YedY is detrimental to the enzyme’s reductase activity and results in an eight-fold decrease in catalytic efficiency. Nonetheless this C-terminal tag does not influence the properties of the molybdenum active site, as assayed by EPR spectroscopy. When a cleavable His-tag was fused to the N-terminus of the mature enzyme in the absence of the signal sequence, YedY was expressed and folded with its cofactor. However, when the signal sequence was added upstream of the N-ter tag, the amount of enzyme produced was approximately ten-fold higher.

****Conclusion**:**

Our study thus underscores the risk of using a C-terminus tagged enzyme while studying YedY, and presents an alternative strategy to express signal sequence-containing enzymes with an N-terminal tag. It brings new insights into molybdoenzyme maturation in *R. sphaeroides* showing that for some enzymes, maturation can occur in the absence of the signal sequence but that its presence is required for high expression of active enzyme.

## Background

Molybdenum-containing enzymes are found in most organisms, and catalyze a wide variety of reactions often involving a two-electron redox chemistry. They are grouped into three separate families, according to the molybdenum cofactor structure and the type of reaction catalyzed: the xanthine oxidase family, the sulfite oxidase family and the DMSO reductase family [1-3].

YedY belongs to the sulfite oxidase family and is found in most Gram-negative bacteria. This enzyme is part of the putative y*edYZ* operon, and was previously isolated and characterized in *Escherichia coli*[4,5]. YedY is a soluble catalytic subunit with a twin-arginine signal peptide required for its export to the periplasm. It contains a Molybdopterin cofactor (MPT), whereas most molybdoenzymes in *E. coli* house a bis(molybdopterin guanine dinucleotide)molybdenum (bis(MGD)Mo) cofactor. YedZ is a membrane-bound cytochrome *b* with 6 putative transmembrane helices, and is probably involved in electron transfer from or to YedY [4]. *E. coli* YedY has been purified and its crystallographic structure was determined at 2.5 Å resolution [5]. The YedY structure reveals that its catalytic domain shares some similarity with chicken liver sulfite oxidase, although the residues involved in the metal coordination sphere are not strictly conserved and the substrate binding sites differ. Moreover, YedY does not exhibit any sulfite oxidase activity, although it can weakly catalyze the reduction of dimethylsulfoxide (DMSO), trimethylamine oxide (TMAO) and L-methionine sulfoxide [5]. Nevertheless, these substrates have a low enzyme affinity (on the order of several tens of mM) suggesting that they are not physiological substrates.

To date, all *E. coli* YedY biochemical studies have been performed using a purified protein labeled with a 6 histidine-tag at its C-terminus [4-6]. His-tag fusion simplifies protein purification, but it may also impair protein expression [7] or be detrimental to either the protein’s function or crystal structure [8]. It is thus advisable to examine expression and activity, either between C- and N-terminal fusions or after tag removal by enzymatic cleavage. N-terminal tagging does have a disadvantage: it is not directly possible with secreted proteins containing a N-terminal signal peptide, since the N-terminal sequence is removed by a specific peptidase upon membrane translocation by the general secretory (Sec) pathway [9,10] or the TAT (twin-arginine translocation) system [11]. The primary role of the twin-arginine pathway is to translocate fully folded proteins across membranes, but it can also participate in protein maturation processes. Redox proteins that have acquired complex multi-atom cofactors in the bacterial cytoplasm are an example of proteins that must be exported in their folded conformation. While it is acknowledged that the TAT signal sequence is essential for protein translocation, as deletion or mutation of this sequence leads to protein accumulation in the cytoplasm [12], its role in protein maturation seems to be protein-dependent. Many TAT-translocated proteins have their own system-specific chaperone, such as TorD (for *E. coli* TMAO reductase) and DmsD (for *E. coli* DMSO reductase), which specifically interact with their partner’s signal sequence [13-15]. Two TorD binding sites are present in the TMAO reductase TorA, with one located near the N-terminal and the other at the core of the protein [3,16]. The DMSO reductase signal sequence is necessary for expression, activity and membrane targeting of the DmsA catalytic subunit. Replacing the DmsA leader with the TMAO reductase TorA leader produces a membrane-bound enzyme with greatly reduced activity and inefficient anaerobic respiration [17]. By contrast, several studies have shown that some active enzymes can be expressed in the absence of the signal sequence, as observed for *E. coli* TMAO reductase [12] or for the heterologous expression of *Rhodobacter sphaeroides* DMSO reductase in *E. coli*[18]. However, enzyme specific activity was not measured in these studies, and how the signal peptide’s absence affects expression level was not quantitatively evaluated. In addition, heterologous expression of *R. sphaeroides* DMSO reductase with its sequence signal in *E. coli* was shown to prevent formation of an active enzyme [18]. Therefore, the TAT signal sequence can be protein-dependent but also species-dependent.

YedY is an intriguing enzyme among the molybdoenzymes. It is widespread and highly conserved, suggesting an important function. However, its role in the cell remains unknown, despite several characterization attempts [4]. Moreover, the x-ray structure of the enzyme in *E. coli* reveals some similarities with the catalytic domain of chicken sulfite oxidase; the residues present in the substrate binding pocket are however more in agreement with a reductase, as confirmed by reductase activity measurements using DMSO or TMAO as the substrates [5]. Despite this, the Km values (on the order of several tens of mM) suggest that the natural substrate has not yet been identified.

In order to identify the YedY substrate, we cloned and expressed *R. sphaeroides* YedY with a 6 His-tag at the C-terminus, using a protocol similar to published studies in the literature with *E. coli* YedY. We observed that the C-terminal tagged enzyme was less active than the native one. We made several constructs to express an enzyme with a removable N-terminal tag in the presence or absence of the signal sequence and compared the kinetics parameters. The results reveal that the C-terminal tag is detrimental to enzyme activity, and that the presence of the signal sequence is important for high expression of the active enzyme.

## Results

### Expression of a C-ter 6 His-tagged YedY from *R. sphaeroides*

**Figure 1:**
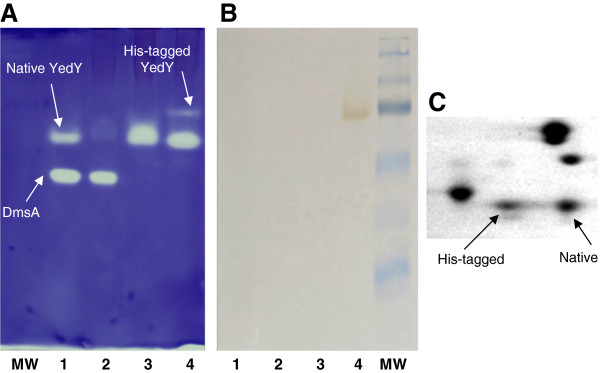
**Comparative DMSO reductase activity of native and C-ter His-tagged YedY.** Non-denaturing PAGE of periplasmic extracts (25 μg) from *R. sphaeroides* f. sp. *denitrificans* WT (lane 1), *yedY*- mutant (lane 2), *dmsA-* mutant (lane 3) and *dmsA-* mutant harboring the pSM88 plasmid (lane 4). **(A)** The gel was stained with dithionite-reduced benzyl viologen and DMSO as substrate; **(B)** Western blot with anti-histidine peroxidase conjugate antibodies; **(C)** A partial 2D PAGE from periplasmic extract of *dmsA-* mutant harboring the pSM88 plasmid, showing spots of native and C-ter tagged YedY (see Additional file 1 for details). MW: molecular weight standards.

The sequence of the molybdenum-containing catalytic subunit YedY is highly conserved in Gram-negative bacteria. YedY from *R. sphaeroides* f. sp. *denitrificans* (34 kDa) and *E. coli* share 50% identical amino acid residues. The *yedY* gene was PCR-amplified from *R. sphaeroides* f. sp. *denitrificans* chromosomal DNA and cloned into pIND4, an IPTG-inducible expression plasmid (developed by Dr. Armitage and colleagues) for protein expression in *R. sphaeroides* and *Paracoccus denitrificans*[19]. The resulting plasmid (pSM88) encodes YedY protein with a 6 histidine-tag at the C-terminus, and was introduced by conjugation into: wild-type *R. sphaeroides*; a *yedY* null mutant (*yedY*^-^); and a DMSO reductase null mutant (*dmsA*^-^) [20]. Periplasmic extracts of wild-type, *yedY*^-^ mutant, *dmsA*^-^ mutant, and *dmsA*^-^ harboring the pSM88 plasmid were separated by non-denaturing electrophoresis (Figure 1). DMSO reductase activity (Figure 1A) was assayed on the gel stained with reduced benzyl viologen, as an electron donor. Two bands are visible in the wild-type extract (lane 1). One band is due to DmsA, the DMSO reductase catalytic subunit which is absent from the *dmsA*^-^ mutant (lane 3), and the other band is due to the DMSO reductase activity of YedY (absent from the *yedY*^-^ mutant, lane 2). In lane 4, the YedY fused to a C-terminal tag (and encoded by the plasmid DNA) has a smaller relative mobility than the native enzyme encoded by chromosomal DNA. We have previously observed for several other proteins, that small changes in amino acid sequence can induce a significant change in protein mobility under these electrophoretic conditions. The western blot (Figure 1B) confirms that this supplementary band is due to the C-ter tagged enzyme. As the activity of the His-tagged protein appeared lower than in the native protein, we checked for the relative amount of protein by 2D electrophoresis. Comparison of the patterns of 2D polyacrylamide gels containing extracts from the *dmsA*^*-*^*mutant*, *dmsA*^*-*^ harboring pSM88 (C-ter tagged YedY) or *dmsA*^*-*^ harboring pSM120 (native YedYZ) strains allowed us to identify the protein spots that are due to the native and His-tagged YedY (Additional file 1). Part of the gel containing extracts from *dmsA*^*-*^ harboring pSM88 is shown in Figure 1C, revealing that the size of native and His-tagged YedY spots are quite similar. Relative amounts of protein (from 2D electrophoresis) and activity staining were estimated with the Genetools program (Syngene). YedY-specific DMSO reductase activity (activity per mg of enzyme) was several-fold lower for the C-terminal His-tagged enzyme than for the native enzyme.

**Figure 2:**
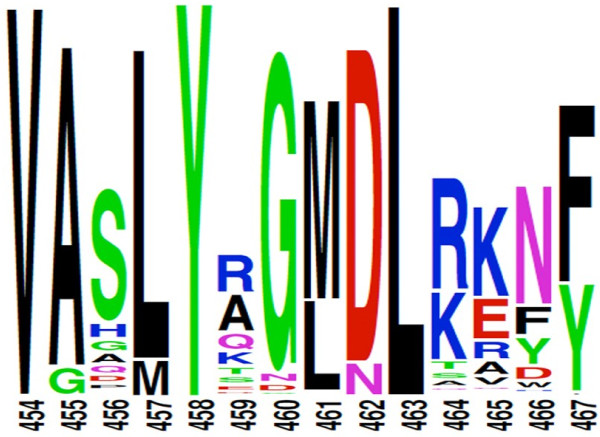
**Weblogo representation of the alignment of C-terminus YedY sequences.** In this representation, the overall height of a stack indicates the sequence conservation at that position (among 1852 sequences), while the height of symbols within the stack indicates the relative frequency of each amino acid at that position [22].

Sequence analysis was then used to examine the basis for the lower activity measured in the C-terminal-labeled enzyme. We performed a PSI-Blast, resulting in 1852 sequences aligned by Clustal X [21]. The conserved pattern obtained for the last 14 residues was visualized with Weblogo [22] (Figure 2). Most of the 1852 sequences end with a highly conserved C-terminal hydrophobic residue (either a phenylalanine or a tyrosine). The *E. coli* YedY crystal structure [5] reveals an asymmetric unit containing five monomers with a disordered C-terminus, in which the last seven residues and the additional 6 histidines are absent from the model. Combined with the qualitative information obtained on the His-tagged protein specific activity, these structural considerations suggest that addition of a hydrophilic affinity tag may destabilize the terminal hydrophobic residues and impair YedY folding and activity. In addition, this could explain the very high Km values that have been obtained for substrates tested on recombinant enzymes [5].

### Strategies for expression of YedY tagged with 6 histidines at the N-terminus

These preliminary results suggested that the C-ter tag may impair folding and activity. To further investigate this, we decided to compare the kinetics parameters of untagged and C-ter tagged purified enzymes.

**Figure 3:**
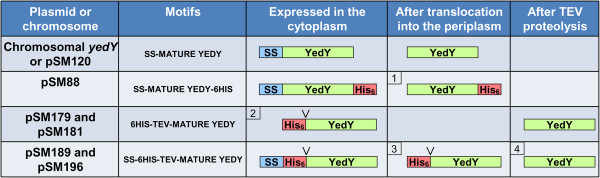
**Constructs of the different YedY enzymes expressed in this study.** The enclosed numbers refer to the proteins studied in this work: (1) corresponds to the C-ter tagged protein (31.4 kDa); (2) is the N-ter tagged enzyme lacking the signal sequence (-SS; 32.6 kDa); (3) is the N-ter tagged protein with the signal sequence (+SS; 32.6 kDa); and (4) is the untagged protein (30.4 kDa). The “V” symbol indicates the position of the TEV protease recognition sequence for cleavage.

For this, we engineered an enzyme with a cleavable His-tag. Since proteases cleave protein substrates downstream of a specific recognition sequence, the tag must be introduced at the N-terminus of the protein to avoid presence of residual amino acids after cleavage. One issue for YedY is that the sequence encoding the 6 His-tag cannot be added upstream of the TAT signal sequence, or else it would be cleaved along with the signal sequence during translocation into the periplasm. On the other hand, the 6 His-tag can be cloned upstream of the sequence encoding the mature enzyme and expressed in the cytoplasm, although absence of the signal sequence may impair protein expression or maturation in some cases [17]. We therefore decided to make two different constructs, one of which contains a 6 His-tag at the N-terminus that is cleavable by the TEV (Tobacco Etch Virus) protease, followed by the mature protein encoding sequence. We used the pET-TEV plasmid [23] which harbors a Ribosome Binding Site (RBS), a 6 His-tag and a TEV (Tobacco Etch Virus) protease recognition site, all upstream of a multiple cloning site. The resulting plasmid (pSM179) contains the motifs RBS-6His-TEV-matureYedY. For the second construct, the TAT signal sequence (SS) was added to the pSM179 plasmid between the RBS and the 6 His-tag coding sequence, resulting in a plasmid (pSM189) harboring the RBS-SS-6His-TEV-matureYedY motifs. Using the PRED-TAT software (Department of Computer Science and Biomedical Informatics, University of Central Greece), we verified that the signal sequence was still recognized as a putative TAT signal sequence and that cleavage after translocation to the periplasm should occur between the SS and the 6 His (which should leave a protein containing 6 His-TEV-matureYedY motifs in the periplasm). The different obtainable forms of YedY enzymes are compiled in Figure 3.

### Heterologous expression in *E. coli*

**Figure 4:**
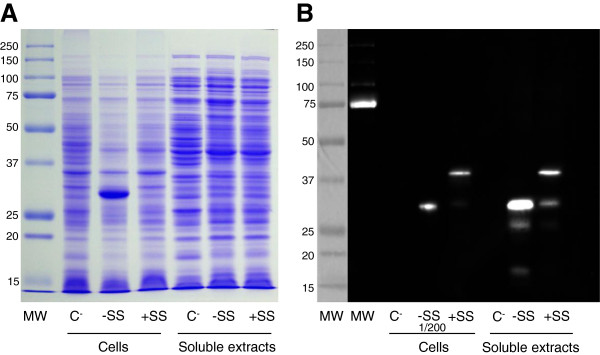
**Influence of the presence of the signal sequence on YedY expression in *E. coli*. (A)** SDS PAGE of whole cell extracts or soluble extracts (25 μg) from *E. coli* BL21 (DE3) that harbor: (lane C-) an empty plasmid (negative control); (lane -SS) the plasmid pSM179 (which contains only the sequence encoding the mature protein); (lane + SS) the plasmid pSM189 (which contains both the mature protein sequence and the signal sequence). **(B)** Western blot analysis of the same samples with anti-histidine peroxidase conjugate antibodies. In lane -SS, the whole cell extract was diluted 200-fold to avoid saturation of the signal. MW: molecular weight standards.

The pSM179 and pSM189 plasmids were introduced into *E. coli* BL21 (DE3). Different growing conditions (e.g. temperature, IPTG concentration and induction time) were evaluated to obtain an optimal expression in soluble extracts. Following this, YedY synthesis was induced with 1 mM IPTG overnight at 16°C. YedY expression for both constructs was compared by western blot analysis after SDS PAGE on whole cell extracts and soluble extracts. In cell extracts, a very high amount of YedY was visible for the construct lacking the signal sequence, even by Coomassie staining (Figure 4A, lane -SS). For western blot analysis, the same sample had to be diluted 200-fold to result in a clearly defined band (Figure 4B, lane Cells -SS). However, the amount on soluble extracts was much lower, as it was not necessary to dilute the sample. These analyses indicate that a high amount of protein is expressed, even though it aggregates in inclusion bodies or in the membrane and only a small part can be detected in soluble extracts. No additional band was visible by Coomassie staining for the construct containing the signal sequence, in comparison to the control, although two bands were detected on the western blot. One band displayed the same relative mobility as the mature protein and most probably corresponds to the protein resulting from signal sequence cleavage (estimated molecular weight 32.6 kDa), while the second band likely corresponds to the protein which still contains its signal sequence (estimated molecular weight 36.6 kDa).

Attempts to purify enzymes from the two cultures resulted in very low protein yield in both cases (less than 1 mg for 6-liter cultures). Despite this, we were able to estimate the reductase activity of both purified proteins by native gel electrophoresis. We could thus determine that the protein resulting from the construct lacking a signal sequence was unable to reduce DMSO, in contrast to the protein with a signal sequence (data not shown). This demonstrates the requirement of the signal sequence for heterologous expression in *E. coli*, as otherwise the protein is synthesized in a very high amount but is inactive and precipitates into inclusion bodies.

### Homologous expression in *R. sphaeroides*

**Figure 5:**
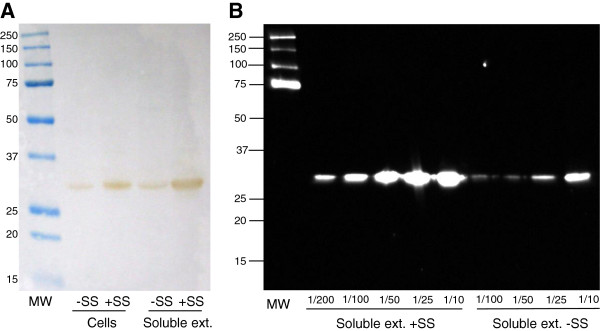
**Influence of the presence of the signal sequence on YedY expression in *R. sphaeroides*. (A)** An image of the membrane after western blot analysis. Whole cell extracts or soluble extracts (25 μg) were loaded on SDS PAGE. Lane -SS: cells harboring the pSM181 plasmid (which contains only the sequence encoding the mature protein). Lane + SS: cells harboring the plasmid pSM196 (which contains the signal sequence upstream of the sequence encoding the mature protein). **(B)** Western blot analysis of the soluble extracts -SS and + SS after different levels of dilution (for example 1/100 represents 100-fold less protein loaded and corresponds to 0.25 μg of protein). Western blot analysis was performed with anti-histidine peroxidase conjugate antibodies.

**Figure 6:**
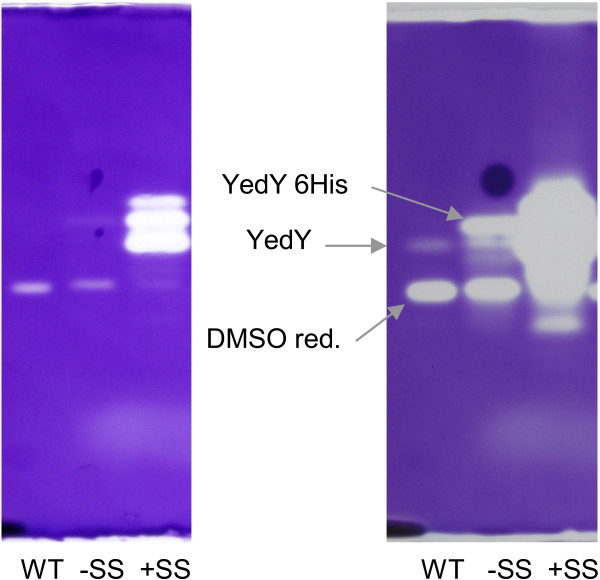
**Influence of the presence of the signal sequence on YedY DMSO reductase activity in *R. sphaeroides.*** Non-denaturing PAGE of soluble extracts (25 μg) from *R. sphaeroides* f. sp. *denitrificans,* stained with dithionite-reduced benzyl viologen and DMSO as substrate. Lane WT: wild-type harboring an empty plasmid. Lane -SS: wild-type harboring the pSM181 plasmid (which contains only the sequence encoding the mature protein with an N-terminal 6 His-tag). Lane + SS: wild-type harboring the plasmid pSM196 (which contains the signal sequence upstream of the sequence encoding the mature protein). Pictures were taken at two different times after addition of substrate. The arrows indicate the bands that correspond to the DMSO reductase DmsA, the chromosome-encoded YedY, and the plasmid-encoded His-tagged YedY.

For expression in *R. sphaeroides* we used the pMS742 replicative plasmid which is a pBBR1MCS-2 derivative [24] containing the promoter of the *puc* operon (encoding the LHII light-harvesting complex). The presence of this strong promoter in the plasmid is routinely used, as it allows the synthesis of high amounts of protein in *R. sphaeroides*[25,26]. The RBS-6His-TEV-matureYedY and RBS-SS-6His-TEV-matureYedY DNA fragments were individually cloned downstream of the *puc* promoter, respectively resulting in pSM181 and pSM196. These plasmids were then introduced into *R. sphaeroides* by conjugation. Cells were grown until the late exponential phase, and whole cells and soluble extracts were separated on SDS PAGE. As shown by western blot, the amount of YedY is higher for the construct with the signal sequence (+SS), both in whole cells and in soluble extracts (Figure 5A). Since the signal on the initial western blot was saturated, the experiment was performed using several sample dilutions (Figure 5B), and the relative amount of the recombinant enzyme in each sample was evaluated with the Genetools (Syngene) Software at several exposure times. YedY was approximately ten-fold more abundant when it was overexpressed with its signal sequence. Contrary to what was observed in *E. coli*, the expressed enzyme that lacks a signal sequence was active (Figure 6). Furthermore, YedY expression with the signal sequence results in a considerably larger DMSO reductase activity. This difference could be attributed to a higher specific activity, or even the difference in YedY relative amount (Figure 5), as further argued below. Another difference with expression in *E. coli* is that when the protein is expressed with the signal sequence, only one band corresponding to the mature enzyme form (32.6 kDa) is visible. Enzyme localization was examined and the protein was observed to be directed to the periplasm when its signal sequence was present (Additional file 2). This result indicates that the signal sequence was recognized by the TAT machinery and that modification of the residues downstream of the sequence (due to addition of the 6 His-tag and the TEV recognition site) did not impair recognition and cleavage.

### Influence of the His-tag and the signal sequence on enzyme properties

**Figure 7:**
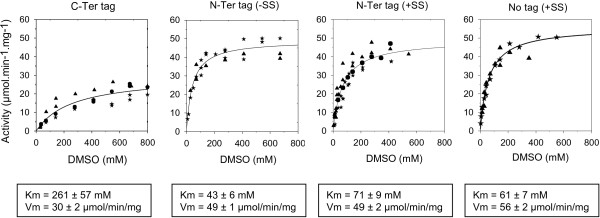
**Kinetics analysis of YedY activity.** DMSO reductase activity of several purified fractions of YedY was measured in a glovebox with reduced benzyl viologen as electron donor and DMSO as substrate. The oxidation of benzyl viologen was spectrophotometrically monitored at 600 nm. The four enzymes were purified from *R. sphaeroides* strains either harboring the plasmid pSM88 for the “C-ter tag” YedY, plasmid pSM181 for the “N-ter tag (-SS)” enzyme or pSM196 for the “N-ter tag (+SS)” enzyme. The enzyme “No tag (+SS)” results from cleavage of the “N-ter tag (+SS)” enzyme with TEV protease. For each plot, at least two experiments with independent biological samples were used. Nonlinear regression of the Michaelis–Menten equation was calculated with SigmaPlot analysis software.

To avoid any competition during molybdenum cofactor incorporation, YedY was purified from the *dmsA*^*-*^ strain; otherwise, as DMSO reductase is quite abundant under these growing conditions, its synthesis can compete for molybdenum cofactor. The enzymes were purified from cells harboring plasmids pSM88, pSM181 and pSM196, and their kinetic parameters were compared. Reductase activity was measured under anaerobic conditions with benzyl viologen as an electron donor and DMSO as a substrate. The initial reaction rates were plotted as a function of DMSO concentration, and the nonlinear regression of the Michaelis–Menten equation was calculated using the SigmaPlot analysis software (Figure 7). The enzyme that is expressed lacking the signal sequence (designed as N-ter tag (-SS)) has a specific activity and an affinity for DMSO similar to those of the enzyme expressed with the signal sequence (N-ter tag (+SS)). This indicates that the difference in activity observed in Figure 6 is due to the difference in YedY amount. Therefore, in the absence of signal sequence, *R. sphaeroides* is able to synthesize a fully active enzyme, although the amount of enzyme is much less (approximately ten-fold).

**Figure 8:**
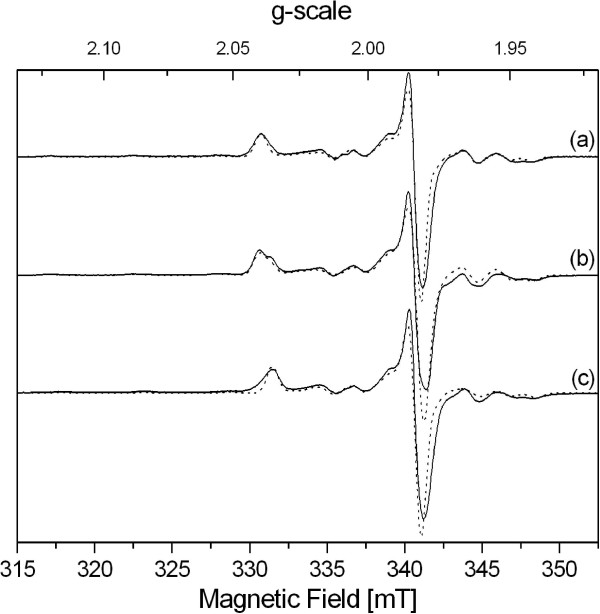
**X-band EPR spectra of as-isolated YedY. (A)** C-ter tagged YedY, **(B)** N-ter tagged YedY and **(C)** YedY after cleavage of the tag. Conditions: T = 55 K, microwave frequency = 9,413 GHz, microwave power = 1 mW, modulation amplitude = 0.5mT. The spectral simulation (dashed lines) yields **(A)** 100% of species 1, **(B)** 67% / 33% of species 1 and 2 respectively, **(C)** 100% of species 2.

The position of the tag was observed to have a dramatic effect on the behavior of catalysts. No significant difference was observed when the tag was fused to the N-terminus (Vm = 49 μmol/min/mg; Km = 71 mM), or removed after its cleavage by TEV protease. By contrast, the catalytic efficiency was eight-fold lower when the tag was fused to the C-terminal hydrophobic residue of the enzyme (Vm = 30 μmol/min/mg; Km = 261 mM). This difference is not due to protein oligomerization state since the elution profile on exclusion chromatography column showed that the three enzymes are monomeric (Additional file 3), as also determined for *E. coli* YedY [5]. In addition, the electron paramagnetic resonance (EPR) signal of YedY molybdenum cofactor was compared for the three purified enzymes. Low-temperature (T = 55 K) X-band (9.4 GHz) EPR spectra are presented in Figure 8 for different preparations of YedY with either an N-ter or C-ter tag, or no tag. Depending on the preparation, the Mo(V) state accounts for 8 to 29% of the total protein concentration. As reported for *E. coli* C-ter His-tagged YedY [6,27], this Mo(V) signal corresponds to the presence of two axial Mo(V) EPR signatures which possess slightly different g- and hyperfine coupling A(^95/97^Mo)- tensors. Simulations of EPR spectra have been performed, revealing that differences between spectra depend only on the ratio of the two species (Table 1); this ratio in turn varies with the preparation. The simulated values are close to those obtained for *E. coli* C-ter His-tagged YedY [6], and therefore no tag influence was observed on the Mo(V) EPR signal. These results indicate that the first coordination sphere of the molybdenum cofactor is not modified, and that the electronic structure of the Mo(V) center appears to be sensitive to preparation and not to the tag position. Such heterogeneity in *R. sphaeroides* YedY samples is comparable to that observed in many other Mo-enzymes [2,28,29]. Nevertheless, the resemblance of the two Mo(V) EPR signatures observed for YedY suggests that the direct Mo(V) environment is almost identical, and that only small structural variations must be present in its coordination sphere.

**Table 1:** EPR simulation parameters for as-isolated YedY

	**g**_**1**_	**g**_**2**_	**g**_**3**_	**A**_**1**_	**A**_**2**_	**A**_**3**_	**α**	**β**	**γ**
species 1	2.034	1.974	1.972	157.0	60.2	60.2	78.6	19.5	-68.8
species 2	2.030	1.973	1.971	157.0	58.8	61.0	79.7	32.1	-84.4

*Hyperfine splitting A*_*i*_*are expressed in MHz and Euler angles (α, β, γ) in degrees*.

## Discussion

YedY is present in most Gram-negative bacteria, and its sequence is highly conserved (50% identical residues between *R. sphaeroides* and *E. coli* enzymes). This suggests an important biological role, yet null mutants in *E. coli*[4] or *Caulobacter crescentus*[30] do not present a marked phenotype. Moreover, even though the purified enzyme is able to reduce some compounds like DMSO or TMAO, affinity for these substrates is quite low (Km values on the order of several tens of mM) and may not reflect the enzyme’s *in vivo* function in bacteria. We constructed a null mutant in *R. sphaeroides* f. sp. *denitrificans* in order to elucidate the perplexing function of this molybdoenzyme. However, this null mutant did not display a clear phenotype (data not shown), as similarly noted in other species. We therefore cloned and purified YedY with a C-terminus 6 His-tag and estimated DMSO reductase activity by non-denaturing gel electrophoresis. Unexpectedly, this activity was lower than the activity due to the native YedY encoded by chromosomal DNA. When activities were compared and correlated to relative amounts of protein (Figure 1 and Additional file 1), it was confirmed that the specific activity of tagged enzyme was several fold lower than for the untagged enzyme. In order to quantify this difference, several plasmids were constructed that can express an enzyme with a cleavable N-terminal tag (Figure 3), and the different enzymes were purified. The three enzymes, either with a C-ter tag, an N-ter tag or no tag (N-ter tag cleaved with TEV protease), were compared for DMSO reductase activity (Figure 7). The N-ter tag does not change kinetic parameters; however the C-ter tag is responsible for an eight-fold decrease in catalytic efficiency that affects both affinity and turnover. This decrease is not due to a change in oligomerization state since the three enzymes are monomeric. EPR spectra (Figure 8) show that the direct Mo(V) environment is not modified by the tag presence; small differences were observed but these are more related to the preparation than to the tag. The detrimental effect of the C-ter tag is most probably due to the structural disorder introduced by the hydrophilic tag fused to the hydrophobic C-terminus tail. Indeed, the crystallographic structure of *E. coli* YedY [5] reveals that the last seven residues and the 6 histidines are disordered. In addition, the last C-terminal helix protrudes away from the protein and is in contact with a non-crystallographic symmetry related monomer. Considering the hydrophobic nature of the C-terminus and the last residue (phenylalanine), this cannot reflect a real arrangement of the untagged enzyme in which the hydrophobic tail is anticipated to fold back on a groove at the surface of the protein.

Our results demonstrate that the C-terminus tail must remain free of additional residues, and that use of a C-ter tag should be avoided when studying YedY properties. Interestingly, all studies to date on YedY have been performed with an enzyme that is not fully active due to this C-ter tag. We have examined several substrates to address this situation, but so far we have been unable to identify one with an improved affinity.

The desire for an N-ter tag directed us to examine the role of the TAT signal sequence in YedY expression. The TAT machinery allows protein translocation to the periplasm, in a fully folded form with inserted cofactors. Several studies have described the existence of a “control” exerted to avoid translocation of unfolded proteins that are consequently degraded [31]. When *R. sphaeroides* YedY is expressed in *E. coli* in the absence of signal sequence, the protein accumulates in an insoluble and inactive form (Figure 4). This could be due to the non-physiologic growth conditions used (overnight incubation at 16°C). However, using these same growth conditions, in the presence of the signal sequence, does not result in any inclusion bodies, and the protein is synthesized in an active form. This demonstrates that the presence of the signal sequence is important to stabilize the enzyme. Even though the signal sequence differs between *E. coli* YedY and *R. sphaeroides* YedY (22% identity), the *R. sphaeroides* signal sequence is necessary for heterologous expression in *E. coli*. Hilton *et al.*[18] obtained a different result for heterologous expression of *R. sphaeroides* DMSO reductase. In their study, they showed (by zymogram and western blot) that the enzyme was synthesized in a high amount in the presence of the signal sequence, even though it was inactive in *E. coli*. By contrast, enzyme synthesis was considerably reduced in the absence of the signal sequence, although an active enzyme was still produced. This shows that the role of the signal sequence in the maturation process can differ from one enzyme to another and is species-dependent. For example, *R. sphaeroides* is able to produce a fully active YedY (Figures 6 and 7) in the absence of its signal sequence, as opposed to *E. coli,* but with a very low yield (approximately 10-fold less than with the signal sequence). This illustrates that the signal sequence is not strictly required for the insertion of the molybdenum cofactor and enzyme folding; on the other hand, it provides critical help in YedY biogenesis, most probably via the involvement of chaperones that protect apoenzyme from proteolysis. Indeed, the REMP (Redox Enzyme Maturation Protein) group of chaperones are involved in maturation and insertion of cofactors for TAT-dependent redox enzymes [32]. They are often found in the same operon as their corresponding gene-encoded TAT substrates. The best characterized ones are TorD and DmsD from *E. coli*; these interact with the signal sequence of their substrate (TorA and DmsA, respectively) as well as with other enzymes [32,33]. Although these chaperones are crucial to obtain a high amount of fully active molybdoenzymes, some active enzyme is still produced in their absence [15]. This is probably what happens when YedY is expressed without its signal sequence: the interaction with putative REMP may be impaired, but a small amount of active enzyme is still produced. No gene encoding a putative specific chaperone has been found in the genomic region of *yedYZ*. It could thus be localized elsewhere in the genome, or YedY maturation could involve some other well-known REMP. Recently, it was shown in *E.coli* that neither DmsD nor TorD are necessary for YedY maturation [34]. Mechanisms and chaperones involved in TAT-dependent translocation for *R. sphaeroides* have not yet been described, and it remains unknown if the maturation of YedY involves a REMP. Nevertheless, among the proteins that co-purified with YedY during its purification, we identified two chaperones by MALDI-TOF that are homologous to GroEL and Trigger Factor (RSP_2311 and RSP_0142, respectively in *R. sphaeroides* 2.4.1; data not shown). Even though these chaperones (as well as DnaK) are not as specific for redox enzymes as REMP, they are involved in the folding of TAT substrates and can bind the TAT signal sequence [35]. These observations suggest they could be involved in the folding and stabilization of YedY.

These results show that the presence of the signal sequence can be crucial for expression of active periplasmic enzymes. It is therefore advisable, when a protein cannot be tagged at the C-terminus, to add a tag at the N-terminus while conserving the original signal sequence. In this study, we propose an easy two step-cloning method in the peT-TEV plasmid [23] to obtain the following construct: RBS-SS-6His-TEV-mature protein. The resulting protein can be folded with its cofactor and translocated into the periplasm, where the signal sequence will be processed by signal peptidase into 6His-TEV-mature protein. The correctly folded, mature protein containing only three additional residues (GHM) at the N-terminus can be easily recovered, following *in vitro* TEV proteolysis.

## Conclusions

Our study makes a case against using a C-ter tagged enzyme when studying YedY, since the presence of the tag at this position affects both the folding and activity of the enzyme. On the other hand we show that maturation of the enzyme can occur in the absence of the TAT signal sequence but that its presence is required for high expression of active enzyme. We propose an easy two-step cloning procedure for expression of an enzyme with a cleavable N-ter tag, all while keeping the signal sequence.

## Methods

### Bacterial strains and growth conditions

Strains and plasmids used in this study are listed in Additional file 4: Table S1.

*Rhodobacter sphaeroides* f. sp. *denitrificans* IL106 was grown at 30°C in 100 ml Hutner medium (Clayton, 1960) in 250 ml Erlenmeyer flasks (150 rpm). When required, either 25 μg/ml kanamycin or 50 μg/ml spectinomycin and 50 μg/ml streptomycin or 1 μg/ml tetracycline were included in the medium. *Escherichia coli* was grown in Luria-Bertani medium. When required, either 25 μg/ml kanamycin or 50 μg/ml spectinomycin and 50 μg/ml streptomycin was included in the medium.

### DNA manipulation and sequence analysis

DNA isolation, plasmid purification and restriction analysis were carried out using standard methods. DNA sequencing was performed by GATC Biotech, France.

### *yedY*^-^ mutant

A 582 bp DNA fragment was PCR-amplified from *R. sphaeroides* f. sp. *denitrificans* chromosomal DNA with primers PstIYed and EcoRIYedrev (Additional file 5: Table S2) and cloned into PCR2.1-TOPO (Invitrogen). The resulting plasmid was digested with PstI and EcoRI and the fragment containing part of the *yedY* gene was cloned into psup202 previously restricted with the same enzymes. The resulting plasmid, unable to replicate into *R. sphaeroides*, was moved from *E. coli* to *R. sphaeroides* by conjugation. In comparison to the whole gene, the *yedY* fragment cloned into psup202 has two fragments of 189 bp and 135 bp missing from the 5′ and 3′ ends, respectively. Following one single crossover event, no entire copy of *yedY* was present on the chromosome. This was verified by PCR, as well as the absence of DMSO reductase YedY activity on non-denaturing polyacrylamide gel electrophoresis (PAGE).

### pBBRpuc plasmid (pMS742)

For protein expression in *R. sphaeroides*, we introduced the strong promoter of the *puc* operon [36] (which encodes the light-harvesting complexes (LHII) of *R. sphaeroides* 2.4.1) into the broad-host-range plasmid pBBR1MCS-2 [24]. The PstI-DraII regulatory region containing the *puc* promoter was then PCR-amplified from the plasmid pPS400 [37] with the primers SacIpuc and RXbaIpuc (Additional file 5: Table S2), and cloned into PCR2.1-TOPO (Invitrogen). The resulting plasmid was digested with XbaI and SacI and the 0.7 kb fragment was cloned into pBBR1MCS-2.

### *yedY* cloning

**Untagged YedYZ**: A 2246 bp DNA fragment containing *yedYZ* (596 bp upstream of the *yedY* start codon) was PCR-amplified from *R. sphaeroides* f. sp. *denitrificans* genomic DNA with the “yedYZ” and “RevyedYZ” primers (Additional file 5: Table S2) and cloned into PCR2.1-TOPO (Invitrogen). The plasmid was subsequently digested with XbaI and HindIII and the fragment was cloned into pBBR1MCS-2. The resulting plasmid (pSM120) allows for the expression of YedY and YedZ under the control of their own promoter.

**C-ter His-tagged YedY**: *yedY* was PCR-amplified from *R. sphaeroides* f. sp. *denitrificans* with the primers pINDyed and RpINDyed (Additional file 5: Table S2). PCR product was cloned into PCR2.1-TOPO (Invitrogen) and subsequently digested with BfuAI and HindIII. The 0.9 kb fragment was cloned into pIND4 [19] previously digested with NcoI and HindIII. The resulting plasmid (pSM88) that encodes YedY with a 6 His-tag at the C-terminus was introduced into *R. sphaeroides* f. sp. *denitrificans* by conjugation.

**N-ter His-tagged YedY**: *yedY* was PCR-amplified from *R. sphaeroides* f. sp. *denitrificans* with the primers petYED and RpetYed (Additional file 5: Table S2). PCR product was cloned into PCR2.1-TOPO (Invitrogen) and digested with NdeI and HindIII. The DNA fragment was cloned into pET-TEV [23] previously digested with the same enzymes. The resulting plasmid (pSM179) encodes the mature form of YedY with a 6 His-tag and the TEV motif ENLYFQ (for cleavage with TEV protease) at the N-terminus. The DNA fragment corresponding to the *yedY* signal sequence was PCR-amplified with the primers YedSS and RYedSS (Additional file 5: Table S2) and cloned into PCR2.1-TOPO. The plasmid was digested with NcoI, and the fragment was cloned in the correct direction into pSM179 previously linearized with NcoI. The subsequent plasmid (pSM189) encodes a protein with a 6 His-tag and a TEV protease motif between the signal sequence and the mature protein sequence. The protein is then cleaved by a signal peptidase (AFA/MGS) after its translocation into the periplasm. For expression in *E. coli*, both plasmids were introduced into *E. coli* BL21(DE3) by standard transformation procedure.

For expression in *R. sphaeroides*, the two plasmids (pSM179 and pSM189) were digested with XbaI and HindIII and cloned into pMS742. The resulting respective plasmids, pSM181 and pSM196, were introduced into *R. sphaeroides* by triparental conjugation [38].

### Nucleotide sequence accession number

The *R. sphaeroides* f. sp. *denitrificans yedY* and *yedZ* sequences were submitted to database under accession number [GenBank:KC601850].

### YedY expression and purification

For expression in *E. coli* BL21(DE3), the culture was induced at OD_600_ = 0.6 with 1 mM IPTG overnight at 16°C in LB medium; soluble or whole cell extracts were prepared as described below. Expression in *R. sphaeroides* f. sp. *denitrificans* was accomplished by growing 6-liter cultures under semi-aerobic conditions in Hutner medium until the late exponential phase. Cells were grown with 1 mM IPTG [19] when the pIND4 derivative vector was used (C-ter tagged YedY), and harvested at the end of the exponential phase.

### Preparation of cell extracts

“Periplasmic extracts” and “cytoplasmic extracts” were prepared using lysozyme, as previously described [39]. For “soluble extracts”, cells were resuspended in HEPES 50 mM (pH 8.0) and lysed with a cell disruptor (One Shot, Constant Systems). The suspension was first centrifuged (7000 g, 10 min) and then ultracentrifuged (200,000 g, 1 h). Protein content was measured with the Coo Protein Assay (Interchim). For “whole cell extracts”, 100 μl of culture (OD_600_ = 1) were centrifuged, and the pellet was resuspended in SDS sample buffer and boiled 10 min at 100°C before loading for gel electrophoresis.

### Purification

6 liters of culture were centrifuged and the pellet was resuspended in 300 ml buffer A [20 mM HEPES pH 8.0, 20% (w/v) sucrose]. 10 ml 0.5 M EDTA was added and after 10 min incubation the suspension was centrifuged at 5000 g for 10 min. The pellet was washed in 150 ml cold water and centrifuged at 5000 g for 20 min. The periplasmic fraction was obtained after incubation for 1 h in 150 ml buffer A containing 1 mg/ml lysozyme. The suspension was then centrifuged for 20 min at 5000 g. The supernatant was centrifuged at 200,000 g to remove cell wall debris, and NaCl was added to the solution to a final concentration of 250 mM.

The periplasmic fraction was loaded on a nickel-charged column (HisTrap column, Amersham) and YedY was eluted by an imidazole step gradient. Gel filtration chromatography was performed using a Superdex 200 10/30 column (Amersham Biosciences) equilibrated with 20 mM HEPES (pH 8.0), 50 mM NaCl. The column was previously size-calibrated using commercial gel filtration standards (Amersham Biosciences).

### Cleavage with tobacco etch virus protease

The enzyme concentration was adjusted to 1 mg/ml in 50 mM HEPES (pH 8.0), 250 mM NaCl. The polyhistidine tag was cleaved off using a His-tagged Tobacco Etch Virus (TEV) protease/YedY mass ratio of 1:100 overnight at 20°C. Untagged YedY was further purified by a second Ni column, equilibrated in the same buffer as the first Ni column. The protein was collected with unbound material.

### Polyacrylamide gel electrophoresis

Proteins were separated by polyacrylamide gel electrophoresis (PAGE) on a 10% acrylamide gel. For non-denaturing conditions, running buffer was 20 mM Tris, 200 mM glycine. For Sodium Dodecyl Sulfate (SDS) PAGE, running buffer was 20 mM Tris, 200 mM glycine, 0.1 mM SDS. For two-dimension gel electrophoresis, a strip of the non-denaturing first-dimension gel was excised and incubated at 60°C for 20 min in SDS sample buffer containing: 60 mM Tris–HCl (pH 6.8), 2% SDS, 40 mM dithiothreitol and 0.02% bromophenol blue. For the second dimension, the strip was placed on top of a 10% polyacrylamide gel and denaturing buffer was used for migration. Molecular weight standards (Precision Plus, All Blue) were purchased from BIO-RAD. For one dimension electrophoresis, 25 μg of protein were loaded while 200 μg were used for 2D-electrophoresis.

### Zymogram

Non-denaturing gel electrophoresis was performed and the gel was incubated anaerobically in degassed MES 100 mM (pH 6.0), 2 mM dithionite-reduced benzyl viologen and 200 mM DMSO.

### Western blot analysis

Proteins were transferred to nitrocellulose membrane (Whatman) after PAGE, and western blots were performed using anti-polyhistidine peroxidase conjugate antibodies (Sigma) and Super Signal WestPico Chemiluminescent Substrate (Interchim), according to the manufacturer’s instructions. Luminescence was detected in a G-Box (Syngene).

### Enzymatic activity

YedY reductase activity was spectrophotometrically assayed at 600 nm using reduced benzyl viologen as the electron donor (ϵ_600_ = 10.4 mM^-1^.cm^-1^) and DMSO as the substrate, in a glovebox workstation (MBRAUN Labstar) flushed with nitrogen. Each reaction mixture (1 ml) contained 100 mM MES (pH 6.0), 0.2 mM benzyl viologen reduced with sodium dithionite, and a variable concentration of DMSO. The initial reaction rates were plotted as a function of DMSO concentration, and the nonlinear regression of the Michaelis–Menten equation was calculated with SigmaPlot analysis software. To determine kinetic parameters, three experiments with two to three independent biological samples were used.

### EPR spectroscopy

X-band EPR spectra were collected at 9.4 GHz using a Bruker ELEXSYS 500E spectrometer fitted with an Oxford Instruments ESR 900 helium flow cryostat. Simulations of the EPR spectra were performed using the Matlab toolbox EasySpin [40].

## Abbreviations

DMSO: Dimethylsulfoxide; TAT: Twin-arginine translocation; SDS PAGE: Sodium dodecyl sulfate polyacrylamide gel electrophoresis; MPT: Molybdopterin; TMAO: Trimethylamine N-oxide; PCR: Polymerase chain reaction; TEV: Tobacco Etch Virus; RBS: Ribosome binding site; His: Histidine.

## Competing interests

The authors declare that they have no competing interests.

## Authors’ contributions

GA participated in molecular biology experiments; SG and SB purified the different YedY enzymes; FB carried out EPR spectroscopy and participated in drafting the manuscript; PA and DP participated in the design of the study, interpretation of the data and helped to draft the manuscript. MS designed the experiments, constructed the mutants and the plasmids, characterized the enzymes and wrote the initial draft of the manuscript. All authors read and approved the final manuscript.

## Supplementary Material

Additional file 1 **Visualization of native and His-tagged YedY on 2D gel electrophoresis of periplasmic extracts from *R. sphaeroides.***
Click here for file

Additional file 2 Influence of the presence of the signal sequence on YedY cellular localization. Click here for file

Additional file 3 Elution profile of purified YedY on gel filtration chromatography. Click here for file

Additional file 4: Table S1 List of strains and plasmids used in this study. Click here for file

Additional file 5: Table S2 List of DNA oligonucleotides used in this study. Click here for file
