# Supplementary material for: Detrimental effect of the 6 His C-terminal tag on YedY enzymatic activity and influence of the TAT signal sequence on YedY synthesis
Source: BMC Biochem. 2013 Nov 1;14:28. doi: 10.1186/1471-2091-14-28 (PMC4228395; doi:10.1186/1471-2091-14-28)
Supplement: Additional file 1 — Visualization of native and His-tagged YedY on 2D gel electrophoresis of periplasmic extracts from R. sphaeroides. [file 1471-2091-14-28-S1.ppt]

## Slide 1
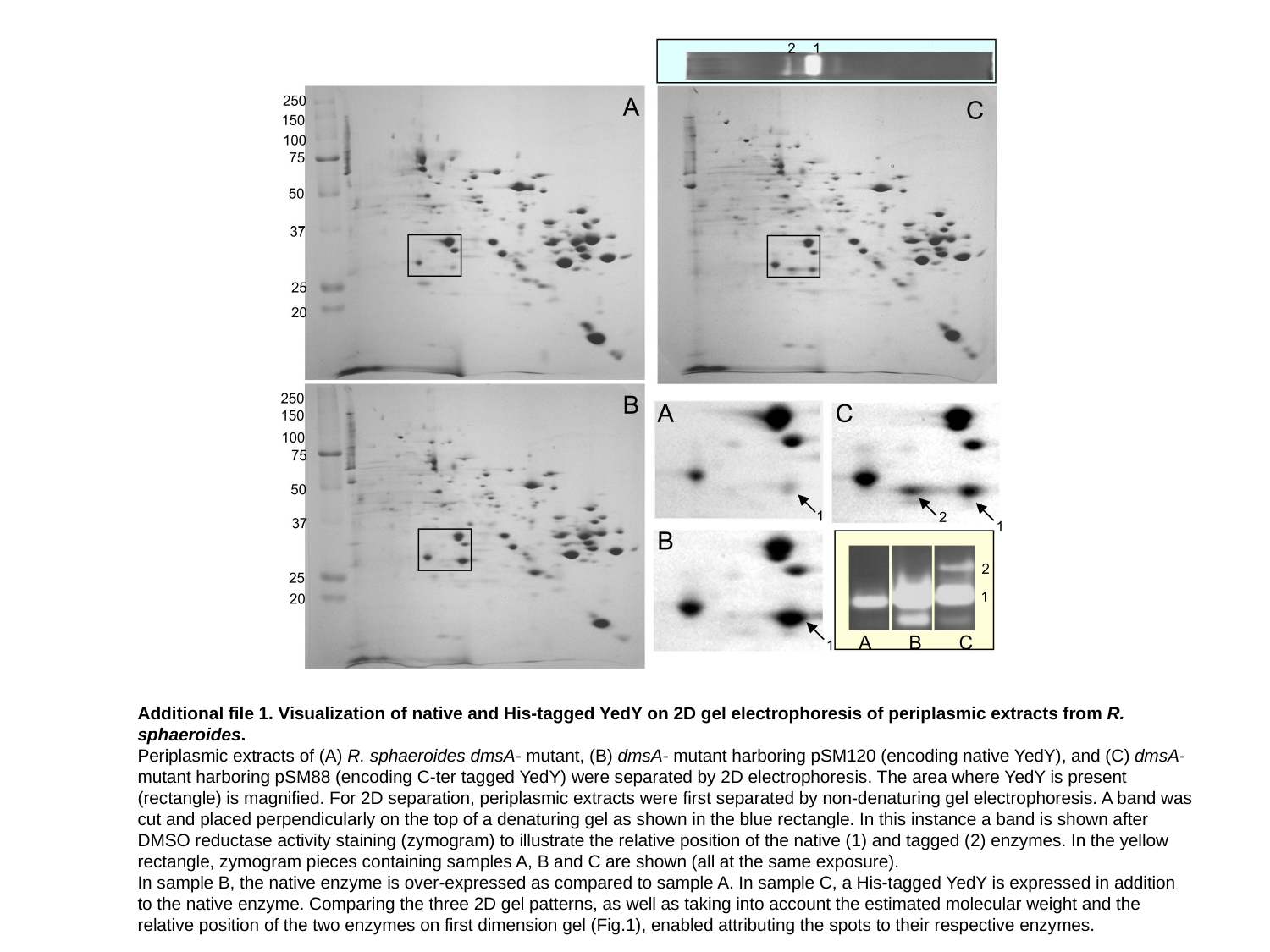

Additional file 1. Visualization of native and His-tagged YedY on 2D gel electrophoresis of periplasmic extracts from R. sphaeroides.
Periplasmic extracts of (A) R. sphaeroides dmsA- mutant, (B) dmsA- mutant harboring pSM120 (encoding native YedY), and (C) dmsA- mutant harboring pSM88 (encoding C-ter tagged YedY) were separated by 2D electrophoresis. The area where YedY is present (rectangle) is magnified. For 2D separation, periplasmic extracts were first separated by non-denaturing gel electrophoresis. A band was cut and placed perpendicularly on the top of a denaturing gel as shown in the blue rectangle. In this instance a band is shown after DMSO reductase activity staining (zymogram) to illustrate the relative position of the native (1) and tagged (2) enzymes. In the yellow rectangle, zymogram pieces containing samples A, B and C are shown (all at the same exposure).
In sample B, the native enzyme is over-expressed as compared to sample A. In sample C, a His-tagged YedY is expressed in addition to the native enzyme. Comparing the three 2D gel patterns, as well as taking into account the estimated molecular weight and the relative position of the two enzymes on first dimension gel (Fig.1), enabled attributing the spots to their respective enzymes.
