# Supplementary material for: Detrimental effect of the 6 His C-terminal tag on YedY enzymatic activity and influence of the TAT signal sequence on YedY synthesis
Source: BMC Biochem. 2013 Nov 1;14:28. doi: 10.1186/1471-2091-14-28 (PMC4228395; doi:10.1186/1471-2091-14-28)
Supplement: Additional file 2 — Influence of the presence of the signal sequence on YedY cellular localization. [file 1471-2091-14-28-S2.ppt]

## Slide 1
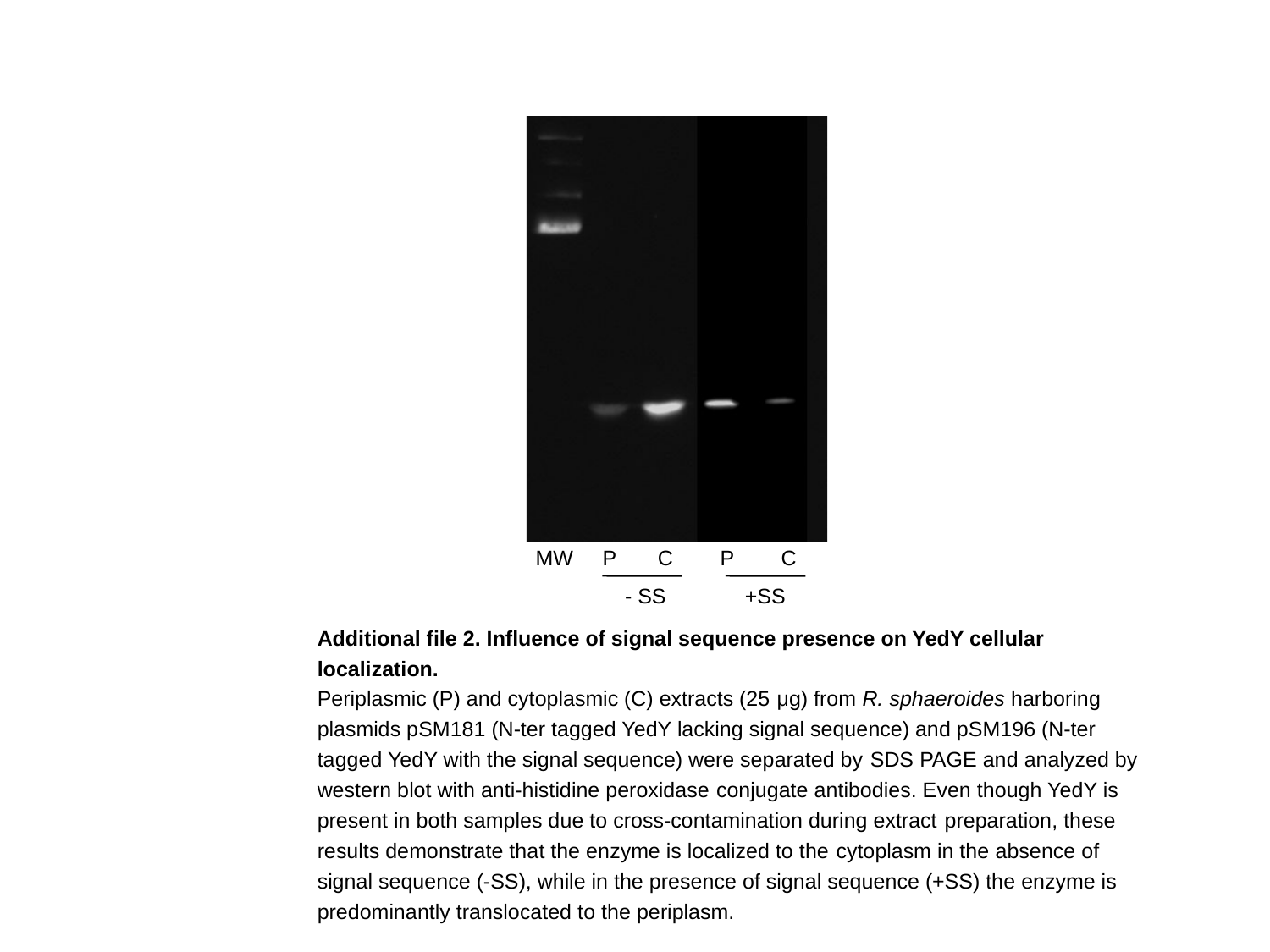

MW P C P C
- SS
+SS
Additional file 2. Influence of signal sequence presence on YedY cellular localization.
Periplasmic (P) and cytoplasmic (C) extracts (25 μg) from R. sphaeroides harboring plasmids pSM181 (N-ter tagged YedY lacking signal sequence) and pSM196 (N-ter tagged YedY with the signal sequence) were separated by SDS PAGE and analyzed by western blot with anti-histidine peroxidase conjugate antibodies. Even though YedY is present in both samples due to cross-contamination during extract preparation, these results demonstrate that the enzyme is localized to the cytoplasm in the absence of signal sequence (-SS), while in the presence of signal sequence (+SS) the enzyme is predominantly translocated to the periplasm.
