# Supplementary material for: Detrimental effect of the 6 His C-terminal tag on YedY enzymatic activity and influence of the TAT signal sequence on YedY synthesis
Source: BMC Biochem. 2013 Nov 1;14:28. doi: 10.1186/1471-2091-14-28 (PMC4228395; doi:10.1186/1471-2091-14-28)
Supplement: Additional file 3 — Elution profile of purified YedY on gel filtration chromatography. [file 1471-2091-14-28-S3.ppt]

## Slide 1
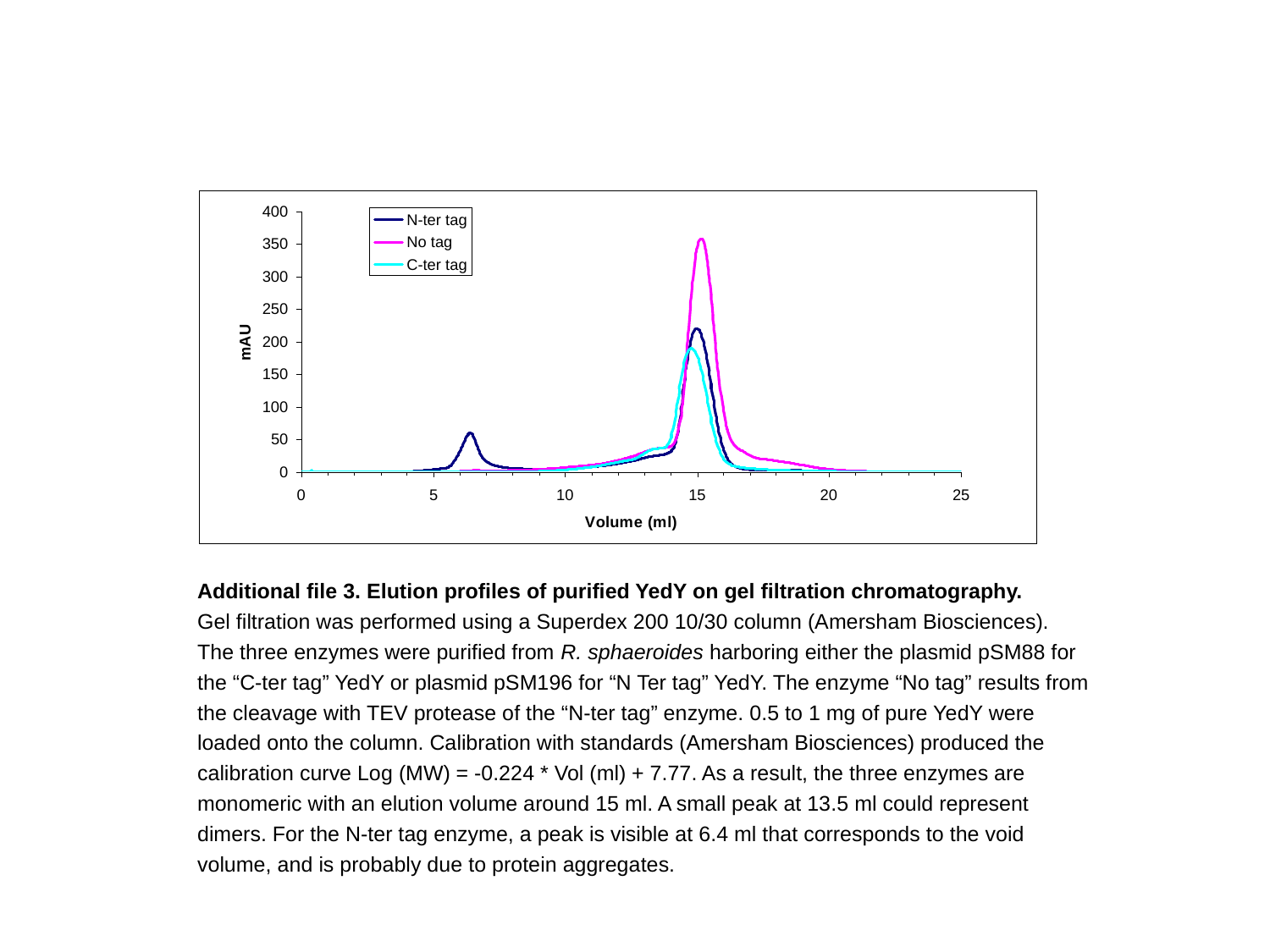

Additional file 3. Elution profiles of purified YedY on gel filtration chromatography.
Gel filtration was performed using a Superdex 200 10/30 column (Amersham Biosciences). The three enzymes were purified from R. sphaeroides harboring either the plasmid pSM88 for the “C-ter tag” YedY or plasmid pSM196 for “N Ter tag” YedY. The enzyme “No tag” results from the cleavage with TEV protease of the “N-ter tag” enzyme. 0.5 to 1 mg of pure YedY were loaded onto the column. Calibration with standards (Amersham Biosciences) produced the calibration curve Log (MW) = -0.224 * Vol (ml) + 7.77. As a result, the three enzymes are monomeric with an elution volume around 15 ml. A small peak at 13.5 ml could represent dimers. For the N-ter tag enzyme, a peak is visible at 6.4 ml that corresponds to the void volume, and is probably due to protein aggregates.
