# Supplementary material for: Detrimental effect of the 6 His C-terminal tag on YedY enzymatic activity and influence of the TAT signal sequence on YedY synthesis
Source: BMC Biochem. 2013 Nov 1;14:28. doi: 10.1186/1471-2091-14-28 (PMC4228395; doi:10.1186/1471-2091-14-28)
Supplement: Additional file 4: Table S1 — List of strains and plasmids used in this study. [file 1471-2091-14-28-S4.doc]

Table S1 : List of strains and plasmids used in this study

| STRAIN OR PLASMID | Genotype or other relevant characteristics | Source |
| --- | --- | --- |
| *E. coli* TOP10 | F′ *mcrA* D(*mrr-hsdRMS-mcrBC*) f80*lacZ*D*M15* D*lacX74 deoR recA1 araD139* D(*ara-leu*)*7697* | Invitrogen |
| *E. coli* BL21(DE3) | F′ *omp*T *hsd* SB(rB-, mB-) *gal dcm* (DE3) | Invitrogen |
| *R.sphaeroides* f.sp.*denitrificans* IL106 |  | [42] |
| *R.sphaeroides* *dmsA-* | *dmsA* null mutant(streptomycine^R^/spectinomycine^R^) | [27] |
| *R.sphaeroides* *yedY-* | *yedY* null mutant, Tet^R^ | This study |
| PLASMID |  |  |
| PCR2.1-TOPO | Cloning vector, Amp^R^, Km^R^ | Invitrogen |
| pET-TEV | pET28a derivative;for protein overproduction in *E. coli,Amp^R^* | [23] |
| pPS400 | pRK415 derivative /0.7 kb PstI-DraII *puc* promoter DNA | [21] |
| pBBR1MCS-2 | Broad-host-range plasmid Km^R^ | [20] |
| pIND4 | Expression plasmid for *R. sphaeroides* Km^R^ | [22] |
| pMS742 | pBBR1MCS2 derivative/ 0.7kb PstI-DraII *puc* promoter DNA | This study |
| pSM120 | pBBR1MCS2 derivative/2.2 kb *yedYZ* fragment | This study |
| pSM88 | pIND4derivative / 6His C-ter tagged YedY | This study |
| pSM179 | pET-TEV derivative / 6His N-Ter tagged Mature YedY | This study |
| pSM189 | as pSM179 with *yedY* signal sequence | This study |
| pSM181 | pMS742 / 6His N-Ter tagged Mature YedY | This study |
| pSM196 | as pMS181 with *yedY* signal sequence | This study |
| psup202 | Broad-host-range mobilization plasmid, Tet^R^ | [43] |
| pSM87 | psup202 + 0.52 kb internal fragment of *yedY* | This study |
