# Supplementary material for: Detrimental effect of the 6 His C-terminal tag on YedY enzymatic activity and influence of the TAT signal sequence on YedY synthesis
Source: BMC Biochem. 2013 Nov 1;14:28. doi: 10.1186/1471-2091-14-28 (PMC4228395; doi:10.1186/1471-2091-14-28)
Supplement: Additional file 5: Table S2 — List of DNA oligonucleotides used in this study. [file 1471-2091-14-28-S5.doc]

Table S2: List of DNA oligonucleotides used in this study

| Primers | 5’🡪3’ sequence |
| --- | --- |
| SacIpuc | CGAGCTCTGCAGGCCCACGCCCTGAA |
| RXbaIpuc | TTCTAGAGTCGGCCTGCACGCGGGCTTC |
| YedYZ | AGATCGACACGCCATTCACC |
| RevYedYZ | TCGGTGAGGCGCTATCTAGG |
| pINDYed | AGACCTGCATATCATGCGCAGGCTCGGATGGTCG |
| RpINDYed | TTAAGCTTGTGATGGTGATGGTGATGGAAATCCACCGAGAGGTC |
| petYed | TCATATGCGGATCGAGGCGAAGGCC |
| RpetYed | GAAGCTTTCAGAAATCCACCGAGAG |
| YedSS | CCATGGGCCGCAGGCTCGGATGGTCG |
| RYedSS | CCATGGCGAAGGCCGGCCCCGCCAGC |
| PstIYed | GCTGCAGTACGAGTTCGGCCTCGACAAG |
| EcoRIYedrev | GGAATTCCGGATGATCCACCGCCGGGTTC |
